# Gemcitabine intercellular diffusion mediated by gap junctions: new implications for cancer therapy

**DOI:** 10.1186/1476-4598-9-141

**Published:** 2010-06-10

**Authors:** Sylvine Cottin, Karim Ghani, Pedro Otavio de Campos-Lima, Manuel Caruso

**Affiliations:** 1 Centre de Recherche en Cancérologie de l'Université Laval, L'Hôtel-Dieu de Québec, Centre Hospitalier Universitaire de Québec, Qc G1R 2J6, Canada

## Abstract

**Background:**

Solid tumors are often poorly vascularized, with cells that can be 100 μm away from blood vessels. These distant cells get less oxygen and nutrients and are exposed to lower doses of chemotherapeutic agents. As gap junctions allow the passage of small molecules between cells, we tested the possibility that the chemotherapeutic agent gemcitabine can diffuse through gap junctions in solid tumors.

**Results:**

We first showed with a dye transfer assay that the glioblastoma and the osteosarcoma cells used in this study have functional gap junctions. These cells were genetically engineered to express the herpes simplex virus thymidine kinase (TK), and induced a "bystander effect" as demonstrated by the killing of TK-negative cells in presence of the nucleoside analogue ganciclovir (GCV). The ability of gemcitabine to induce a similar bystander effect was then tested by mixing cells treated with 3 μM gemcitabine for 24 hours with untreated cells at different ratios. In all cell lines tested, bystander cells were killed with ratios containing as low as 5% treated cells, and this toxic effect was reduced in presence of α-glycyrrhetinic acid (AGA), a specific gap junction inhibitor. We also showed that a 2- or a 24-hour gemcitabine treatment was more efficient to inhibit the growth of spheroids with functional gap junctions as compared to the same treatment made in presence of AGA. Finally, after a 24-hour gemcitabine treatment, the cell viability in spheroids was reduced by 92% as opposed to 51% in presence of AGA.

**Conclusion:**

These results indicate that gemcitabine-mediated toxicity can diffuse through gap junctions, and they suggest that gemcitabine treatment could be more efficient for treating solid tumors that display gap junctions. The presence of these cellular channels could be used to predict the responsiveness to this nucleoside analogue therapy.

## Introduction

Nucleoside analogues are drugs commonly used in the clinic as antiviral and anticancer agents. Gemcitabine (2', 2'-difluorodeoxycytidine, dFdC; Gemzar^®^) is a pyrimidine analogue that has a well established place in the treatment of several types of solid tumors; it is indicated as a single agent for the treatment of metastatic pancreatic cancer and, in combination regimens, for the treatment of non-small cell lung carcinoma, ovarian and breast cancer [1]. The use of gemcitabine is currently being tested in bladder cancer, mesothelioma and head and neck cancer; there are also promising results with the combination of gemcitabine and radiation therapy for the treatment of glioblastoma [1-4].

Gemcitabine enters cells by interacting with transmembrane glycoproteins that control the inward/outward flow of natural nucleosides. The human nucleoside transporters (hNT) are divided into two groups: the equilibrative (hENT) and the concentrative (hCNT) types [5]. Gemcitabine is a good permeant for hENT1, hENT2, hCNT1 and hCNT3. However, hENT1 seems to be the major gemcitabine transporter [5-9].

After its entry into the cell, gemcitabine is converted by the deoxycytidine kinase (dCK) into 2',2'-difluorodeoxycytidine monophosphate which becomes subsequently phosphorylated to the cytotoxic 5'-diphosphate and 5'-triphosphate derivatives by pyrimidine monophosphate and diphosphate kinases. The diphosphate molecule is capable of inhibiting the ribonucleotide reductase (RR) directly; on the other hand the triphosphate molecule is incorporated into DNA and RNA, and affects their synthesis by chain termination [10-12]. The dCK has a higher affinity for gemcitabine than other substrates which results in a more efficient intracellular drug accumulation and toxicity [13]. The above scenario is supported by *in vitro *studies that suggest a correlation between resistance to gemcitabine and the expression level of hENT1, dCK and RR [14]. In addition, patients with non-small cell lung carcinoma and pancreatic cancer that expressed high levels of hENT1 responded better to gemcitabine and survived longer [15-18]. In one study, hCNT3 was also a predictive survival factor after adjuvant gemcitabine therapy in resected pancreatic adenocarcinoma [18].

One limitation to gemcitabine efficacy, that is common to other chemotherapeutic agents, is its poor penetration in solid tumors [19-21]. Gemcitabine is normally administered systemically and reaches the cancer site through blood vessels. The vasculature in solid tumors is poorly organized as compared to normal tissues. Neoplastic cells can be as far as 100 μm distant from the nearest blood vessel, and this results in a gradient distribution of the chemotherapeutic agent. Therefore, tumor cells that are far from the blood supply not only are less exposed to the drug but also tend to be more quiescent due to hypoxia and lack of nutrients, and are intrinsically less sensitive to chemotherapy [19,22]. This is illustrated by the lack of efficacy of gemcitabine treatment in a transgenic mouse model of pancreatic cancer due to the poor vascularization of the tumor [23].

Gap junctions are composed of a family of proteins called connexins that allow passive diffusion of small molecules (≤1 kD) between cells. Ions, short peptides and most second messengers such as cAMP, calcium and innositol 1,4,5-triphosphate can traffic across these molecular channels [24]. It is well documented that ganciclovir (GCV) and its phosphorylated metabolites can diffuse in tumors through gap junctions producing a phenomenon called the "bystander effect" [25,26]. GCV is a nucleoside analogue that is used primarily as an antiviral agent but that may turn into an anticancer drug if tumor cells are engineered to express the herpes simplex virus thymidine kinase (TK) [26,27].

We hypothesize that gemcitabine and its metabolites can diffuse through gap junctions in a way that is reminiscent of the GCV intercellular diffusion. In this study, we show that gemcitabine can induce a bystander effect in glioblastoma and osteosarcoma cells. This effect was demonstrated in two and three-dimensional culture models and was blocked by a specific gap junction inhibitor.

## Materials and methods

### Cell lines and Monolayer Culture

The human glioblastoma cell line U87 (ATCC HTB-14), and the human osteosarcoma cell lines MNNG/HOS (ATCC CRL-1547) and MG-63 (ATCC CRL-1427) were obtained from the American Type Culture Collection. The glioblastoma SKI-1 cell line was obtained from Jacques Galipeau (McGill University, Montreal, Canada). All four cell lines were negative by Hoechst staining for the presence of mycoplasmas. Cells were cultured in Dulbecco's Modified Eagle medium (DMEM; Sigma, St-Louis, MO, USA) supplemented with 10% fetal calf serum (FCS; PAA laboratories, Etobicoke, Canada) and antibiotics. All cultures were maintained in humidified atmosphere with 5% CO_2 _in air at 37°C. Cells containing the GFP/TK gene were generated by retroviral infections [28], and were cultured as their parental counterparts.

### Multicellular Spheroid Culture

Spheroids of SKI-1 and MG-63 cells were generated by the liquid overlay culture technique as previously described [29]. Single-cell suspensions (4 × 10^3 ^or 2 × 10^3 ^cells per well) obtained from exponentially growing monolayer cultures were seeded in 96-well plates coated with a thin layer (50 μl per well) of 1.5% agarose solution, mixed in 1:1 ratio with DMEM. Both cell lines formed well-rounded, regularly shaped spheroids within 3 days of static incubation. At this stage, diameters of spheroids were between 300 and 400 μm.

### Cx43 Immunofluorescence

Cells were cultured on glass coverslips in 35-mm dishes until they reached sub-confluency. Cells were fixed in 4% paraformaldehyde, permeabilized with 0.2% Triton X-100 and incubated with a primary antibody raised against Cx43 (1:800; Sigma) followed by an Alexa594-conjugated goat anti-mouse (1:1000) (Invitrogen). Nuclei were counterstained with Hoechst reagent. Cells were observed with a Bio-Rad MRC-1024 confocal microscope mounted on a Nikon Diaphot-TMD.

### Gap Junctional Intercellular Communication Assay

In monolayer cultures, gap-junctional coupling was measured by double-dye flow cytometry as described previously [28,30]. Briefly, cells were labelled with 3 μM calcein-AM (acetoxymethyl ester) or 5 μM DiI (Invitrogen) diluted in Opti-MEM medium (Invitrogen). SKI-1, MNNG/HOS and MG-63 cells were plated at 0.6 × 10^6 ^cells per cm^2^, and U87 cells at 0.8 × 10^6 ^cells per cm^2 ^to allow intercellular contact. Next, calcein-loaded cells were plated on top of the DiI-stained cells at a ratio of 1:10 (donors:recipients). After 6 hours of incubation at 37°C, the cell mixtures were detached and analyzed by flow cytometry. Fluorescence was measured with a Coulter EPICS XL-MCL flow cytometer and Expo32 software (Beckman Coulter, Brea, CA). Similar dye transfer experiments were performed with the gap junction inhibitor α-glycyrrhetinic acid (AGA; Sigma) [31]. Cells were incubated with AGA at 70 μM 24 hours before the labelling of the cells and during the rest of the experiment. The effect of gemcitabine (kindly provided from hospital Hôtel-Dieu de Québec) on gap junctional intercellular communication was assessed by incubating cells with 3 μM gemcitabine 24 hours before the labelling of the cells and during the rest of the experiment.

Gap junctional intercellular communication was also assessed in three-dimensional cultures. SKI-1 and MG-63 spheroids grown for 4 days in 96-well plates were labelled for 1 hour by replacing 50% of culture medium with a 3 μM calcein-AM solution. Twenty spheroids per condition were then washed with PBS and trypsinized with a 0.25% or a 0.05% solution of trypsine/EDTA, for SKI-1 and MG-63 spheroids, respectively. Dissociated cells were pooled, centrifugated, resuspended in PBS and analyzed for fluorescence by flow cytometry. This experiment was also carried out in presence of 70 μM AGA started 24 hours before and during labelling.

### Gemcitabine Dose Response

Cells were plated in 96-well plates at 10^3 ^cells per well and treated the following day with an increasing concentration of gemcitabine varying from 0.1 nM to 10 μM for 24 hours. The cytotoxic effect of gemcitabine was determined by 3-(4,5-dimethylthiazol-2-yl)-2,5-diphenyltetrazolium bromide (MTT) assay 3 to 4 days later at the time untreated cells reached confluency.

### TK/GCV, Gemcitabine, Cisplatine and Temozolomide Bystander Effect

TK/GCV bystander effect experiment has been described previously [28,32]. Briefly, 10% or 2% TK-expressing cells were mixed and plated in 6-well plates with their respective parental cell lines, at identical concentrations to the ones used for the gap junctional intercellular communication assay. The following day, confluent cells were treated with 10 μM GCV. On day 3, cells were trypsinized and a 1:100 dilution of the cells was distributed into 96-well plates in five replicates. Cells were cultured subsequently in the presence of GCV for 3 days and cell proliferation was measured using the MTT assay. A final concentration of 0.5 mg/ml of MTT was added to wells and the plates were incubated at 37°C for 2 hours. After medium removal, 150 μl of dimethyl sulfoxide was added and the plates were gently shaken for 10 min to dissolve the formazan blue crystals. The absorbance was then measured at 595 nm with a microplate reader (Tecan, Research Triangle Park, NC).

For the gemcitabine bystander effect experiments, cells were plated in 6-well plates at concentrations identical to those used for the dye transfer. The following day, confluent cells were left untreated or were treated with 3 μM gemcitabine for 24 hours. On day 3, treated and untreated cells were mixed at different ratios containing 1, 5, 10 or 50% treated cells, and plated in 24-well plates in order to allow cell-cell contact for 24 hours. The next day, cells were detached and a 1:100 dilution of the cells was disposed in 96-well plates, and cultured for 3 days. Cell proliferation was measured by the MTT assay at day 7. Experiments were also carried out in presence of 70 μM AGA. Similar experiments were performed with 10 μM cisplatine and 1 mM temozolomide.

### Gemcitabine Cytotoxicity in Spheroids

Three days after cell seeding, SKI-1 and MG-63 spheroids were treated with 3 μM gemcitabine for 2 hours or 24 hours in presence or absence of AGA. After the removal of gemcitabine, the spheroids were grown for 6 days and the gemcitabine cytotoxic effect was evaluated by measuring the diameter of each spheroid with a calibrated TE2000 microscope (Nikon, Melville, NY) using Metavue software (Molecular Devices, Sunnyvale, CA). AGA treatment was kept until diameters were measured.

A modified acid phosphatase assay was used to determine cell viability in spheroids [33]. Six days after gemcitabine treatment, each individual spheroid was transferred with supernatant into standard 96-well plates and centrifuged for 10 min at 1, 000 × g. The supernatant was then carefully removed and spheroids were washed with PBS. Plates were once again centrifuged and the supernatant discarded. Next, 100 μl of PBS with 100 μl of the assay buffer containing 0.1 M sodium acetate, 0.1% Triton X-100 and 5 mM *p*-nitrophenyl phosphate (Sigma) was added in each well and incubated for 90 min at 37°C. Finally, 10 μl of 1 N NaOH was added to each well, and the absorbance was measured at 415 nm.

## Results

### Gap junction expression and functionality in glioblastoma and osteosarcoma cells

**Figure 1:**
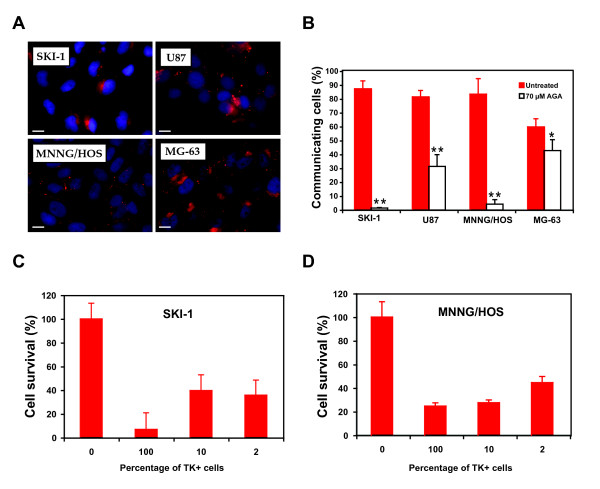
**Cx43 expression and functionality in glioblastoma and osteosarcoma cell lines**. **A**. Immunofluorescence of Cx43 shown by confocal microscopy; scale bar: 20 μm: **B**. Intercellular communication measured by double dye flow cytometry. The percentage of communicating cells represents the percentage of DiI-stained cells that picked up calcein from calcein-loaded cells in presence (open bars) or not of AGA (red bars). Each value is the mean ± s.d. of triplicates of at least three separate experiments. Statistical significance between untreated and AGA-treated cells was evaluated by a Student *t*-test (*, p < 0.05; **, p < 0.01). Bystander effect of the thymidine kinase/ganciclovir strategy: **C**. In SKI-1 glioma cells. **D**. In MNNG/HOS osteosarcoma cells. Data are the means ± S.D. of five replicates of three separate experiments.

The role of gap junctions was tested in two types of cancer that have been previously shown to be responsive to gemcitabine [2-4,34,35]. First, we have analyzed the connexin 43 (Cx43) expression profile in two glioblastoma and two osteosarcoma cell lines. Figure 1A shows that in agreement with our previous report [28], SKI-1 and U87 glioblastoma cells predominantly express Cx43 in cytoplasmic perinuclear compartments. Instead, Cx43 was localized on the plasma membrane as streaks and spots representative of gap-junctional plaques in the osteosarcoma cell line MNNG/HOS. Like SKI-I and U87 cells, MG-63 cells had Cx43 located mainly in cytoplasmic areas, with very little Cx43 at the cell surface (Figure 1A).

Next, the gap junctional intercellular communication was evaluated in these four cell lines by a double-dye flow cytometry assay using ratios of 10% calcein-AM-loaded cells and 90% Dil-labelled cells. The percentage diffusion of calcein into Dil-labelled cells in these experiments was 87.3% for SKI-1 cells and 81.5% for U87 cells. The MNNG/HOS cells were also extensively coupled since calcein diffused into 83.5% DiI-labelled cells. Coupling was less pronounced in MG-63 cells as calcein diffusion reached 59.9% of the neighboring cells. Similar experiments were performed in presence of AGA, a specific gap junction inhibitor; dye transfer in MNNG/HOS and SKI-1 cells were completely abolished in these conditions. Treatment of U87 and MG-63 cells with AGA inhibited the gap junction intercellular communication by 50% and 17%, respectively (Figure 1B). Once we have ascertained that the four cell lines used in this study possess highly functional gap junctions, we have tested their ability to mediate a bystander effect

Diffusion of phosphorylated GCV was studied in the SKI-1 and MNNG/HOS cell lines. First, stable cells expressing GFP/TK were derived from each parental cell line by retroviral gene delivery as described previously [28]. Cell viability was measured after mixing 2% and 10% TK-expressing cells with their parental counterparts followed by GCV treatment. Cell survival was only 40% and 41% of the control in mixtures that contained 10% and 2% TK-expressing SKI-1 cells, respectively (Figure 1C). The cell viability of MNNG/HOS cells was 27.6% and 44.6% in the mixtures that included 10% and 2% TK-expressing cells, respectively (Figure 1D). Our results showed that both cell lines were able to mediate a strong bystander effect, most likely due to the transfer of phosphorylated GCV from TK-expressing cells into TK-negative bystander cells.

### Gemcitabine cytotoxicity

**Figure 2:**
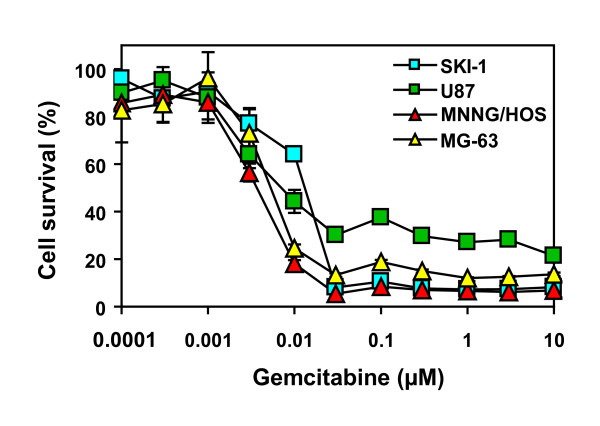
**Gemcitabine cytotoxicity on glioblastoma and osteosarcoma cells**. Cell survival was measured 4 days after a 24-hour gemcitabine treatment. Cell survival was expressed in comparison to untreated cells. Each value is the mean ± S.D. of five replicates.

Before testing the gemcitabine-mediated bystander effect, the sensitivity to a 24-hour drug treatment was assessed in the four gap junction-positive cell lines. All cell lines were quite sensitive to gemcitabine, with concentrations that inhibited cell proliferation by 50% ranging from 3.5 to 13 nM (Figure 2). A dose of 3 μM gemcitabine, that is achievable in the serum of treated patients, was chosen for the following experiments [36,37].

### Inhibition of gap junctional intercellular communication by gemcitabine

**Figure 3:**
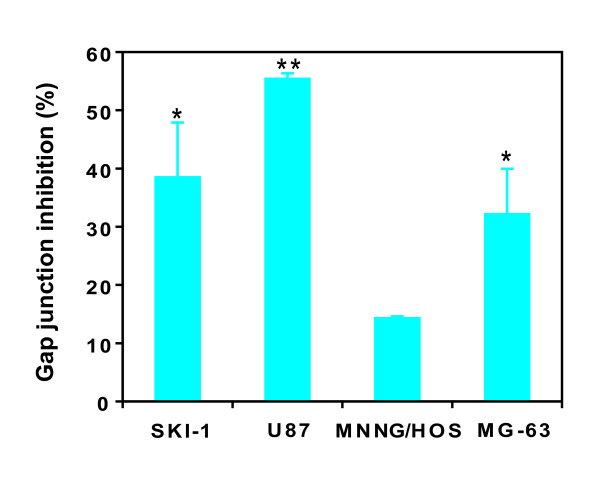
**Inhibiton of dye transfer by gemcitabine**. The transfer of calcein between cells was evaluated after a 24-hour treatment with 3 μM gemcitabine. Data are the means ± S.D. of three independent experiments. Statistical significance for the inhibition of gap junction was evaluated by a Student *t*-test (*, p < 0.05; **, p < 0.01).

The effect of gemcitabine on gap junction functionality was examined because its mechanism of action involves the inhibition of DNA and RNA synthesis [1], and Cx43 has a short half-life [24,38]. Calcein diffusion was measured in both glioblastoma and osteosarcoma cell lines after gemcitabine treatment. Gap junction-mediated dye transfer was inhibited in all cell lines tested, although at different intensities. Reductions of 38.5%, 55.4% and 32.1% were observed in SKI-1, U87 and MG-63 cell lines, respectively. The inhibition was less pronounced in MNNG/HOS cells, with a 14.3% reduction in calcein transfer obtained after gemcitabine treatment (Figure 3).

### Gemcitabine bystander effect

**Figure 4:**
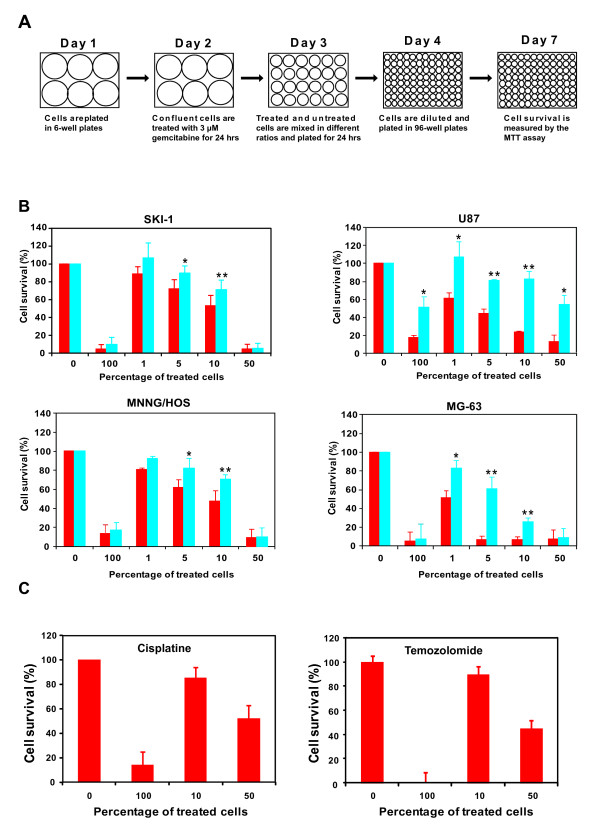
**Bystander effect of gemcitabine in glioblastoma and osteosarcoma cell lines**. **A**. Experimental design of the bystander effect assay: **B**. Bystander effect of gemcitabine with (blue bars) or without AGA (red bars). Data are the means ± S.D. of five replicates of at least three separate experiments. Statistical significance between untreated and AGA-treated cells was evaluated by a Student *t*-test (*, p < 0.05; **, p < 0.01): **C**. Absence of cisplatine and temozolomide bystander effect in SKI-1 and MNNG/HOS, respectively.

The bystander effect mediated by gemcitabine was evaluated in the four cell lines characterized previously. Cells that had been treated with gemcitabine were mixed at different ratios with their untreated counterparts and plated at confluency (after the 24-hour gemcitabine treatment, the number of cells in the treated wells was 50% lower as compared to the untreated wells; treated and untreated cells were equally viable as measured by trypan blue exclusion). After 1 day of contact, cells were diluted and cultured until their viability was measured (Figure 4A illustrates the experimental design). The experiment was also performed in presence of AGA in order to evaluate the involvement of gap-junctions in the bystander effect. All cell lines showed a strong bystander effect since as little as 1% treated cells were able to affect the viability of untreated cells. At this ratio (1/100), the viability was within the 88%-51% range. The viability dropped to even lower levels when the percentage of treated cells used in the mixtures increased. As little as 4% live cells were obtained with a 1/1 treated/untreated ratio in SKI-1 cells. Similar results were achieved with MG-63 cells (7% at 1/1 ratio). The bystander effect was most effective in MG-63 cells: the viability at the 1/100 ratio was 51%, and it decreased to 7% in the 1/20 ratio. In the four cell lines, the bystander effect was significantly inhibited in the presence of AGA at ratios containing 1/20 and 1/10 gemcitabine treated cells. AGA was also capable of inhibiting the bystander effect in U87 and MG-63 cells when the 1/100 ratio was adopted (Figure 4B). These results indicated that the osteosarcoma and glioblastoma cells are susceptible to a gemcitabine-mediated bystander effect that can be blocked by the gap junction inhibitor AGA. On the contrary, there was no gemcitabine-mediated bystander effect with HeLa cells that are devoid of gap junction (data not shown), and there was also no bystander effect with the chemotherapeutic agents cisplatine and temozolomide tested on SKI-1 and MNNG/HOS cells (Figure 4C).

### Gap junctional intercellular communication in spheroids

**Figure 5:**
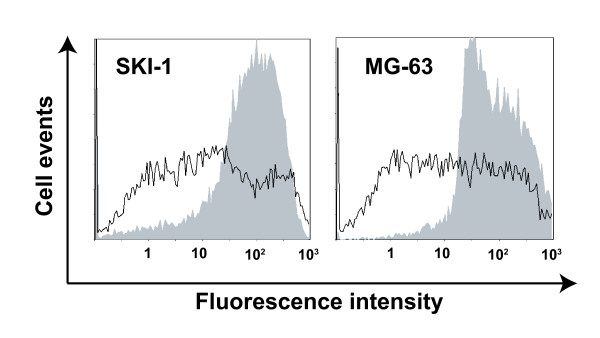
**Dye transfer in multicellular spheroids**. The transfer of calcein was measured in SKI-1 and MG-63 spheroids in absence (grey area) or presence (black line) of AGA. The plot presented for each cell line is representative of an experiment performed three times.

A three-dimensional spheroid culture system was set-up to test the gemcitabine bystander effect in a physiologically more relevant *in vitro *model. First, the diffusion of calcein was evaluated in SKI-1 and MG-63 multicellular tumor spheroids. After one hour labelling with calcein-AM, spheroids were dissociated into single cells and were analyzed for fluorescence by flow cytometry. One sharp highly fluorescent peak was obtained indicating that calcein had diffused well in the spheroid model and that cells had been homogenously labelled. When the experiment was performed in presence of AGA, cells were heterogeneously labelled and has a mean fluorescence intensity that was ten times lower as compared to untreated control cells (Figure 5). These results suggested that gap junctions were functional in the spheroid model and that they could be inhibited by AGA treatment.

### Gemcitabine effect on spheroid growth

**Figure 6:**
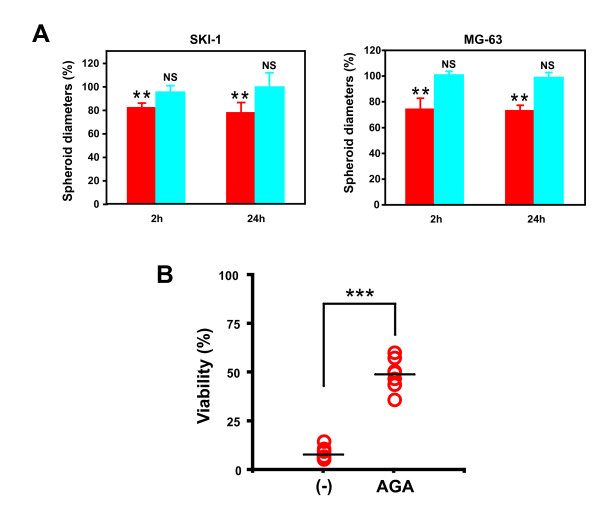
**Gemcitabine cytotoxic effect on spheroids**. **A**. Spheroid diameters were measured 6 days after gemcitabine treatment for 2 hours or 24 hours with (blue bars) or without AGA (red bars). Spheroid diameters are expressed as percentage of untreated spheroids with or without AGA. Data are the means of five replicates ± S.D. of one representative experiment performed twice. Statistical significance between untreated and gemcitabine-treated cells was evaluated by a Student *t*-test (**, p < 0.01; NS, p > 0.05): **B**. SKI-1 viability in spheroids was measured 6 days after gemcitabine treatment for 24 hours. Spheroid viability is expressed as percentage of untreated spheroids with or without AGA (-). Individual data of eight replicates are displayed for each condition. Means are presented as horizontal bars. Statistical significance between untreated (-) and AGA-treated cells was evaluated by a Student *t*-test (***, p < 0.0001).

After having demonstrated that gemcitabine induces a gap junction-mediated bystander effect in standard two-dimensional cell culture systems, we have tested if this effect was also present in tumor spheroids. Gemcitabine treatment for 2 hours was sufficient to inhibit the growth of SKI-1 and MG-63 spheroids by 19% and 27%, respectively (mean diameter ± SD for SKI-1 cells: untreated, 609 ± 33 nm; treated, 497 ± 11 nm; mean diameter ± SD for MG-63 cells: untreated, 483 ± 15 nm; treated, 357 ± 8 nm). The growth inhibition was slightly increased if the gemcitabine treatment lasted 24 hours. Indeed, the volumes were reduced by 23% and 28% in SKI-1 and MG-63 spheroids, respectively (mean diameter ± SD for treated cells: 471 ± 13 nm in SKI-1 cells and 351 ± 12 nm in MG-63 cells). The effect of gemcitabine on spheroids was abolished if the same experiment was performed in presence of AGA, (Figure 6A). These results suggested that functional gap junctions mediate the cytotoxicity of gemcitabine in three-dimensional *in vitro *models of glioblastoma and osteosarcoma.

The effect of gemcitabine on spheroids was next tested with the acid phosphatase viability assay since the measure of spheroid diameters does not distinguish between dead and live cells. Six days after a 24-hour gemcitabine treatment, the cell viability of SKI-1 spheroids was reduced by 92%. In presence of AGA, the viability was only reduced by 51% (Figure 6B). These results further support a role for gap junctions in the gemcitabine-mediated toxicity in three-dimensional in *vitro *models and are in agreement with the data obtained by measuring spheroid diameters.

## Discussion

In this study, we have tested the hypothesis that the chemotherapeutic agent gemcitabine could be more efficient to treat tumors displaying functional gap junctions. We have shown in glioblastoma as well as in osteosarcoma that gemcitabine-treated cells could kill untreated cells in a gap junction-dependent manner.

Both glioblastoma cell lines and MG-63 cells had Cx43 mainly located in cytoplasmic areas with only few plaques at the cell surface. Despite this aberrant localization, these cells could transfer calcein as efficiently as MNNG/HOS cells that had a high level of Cx43 assembled into punctate gap junction plaques (Figures 1A and 1B). It is conceivable that other connexin family members could contribute to gap junctional intercellular communication. However, this possibility would not affect the conclusion of our study that links the functionality of gap junctions and not its composition to the gemcitabine-induced bystander effect.

A strong bystander effect that was responsive to AGA was observed in monolayer cultures in cell mixtures containing 1%, 5% and 10% gemcitabine-treated cells (1/100, 1/20 and 1/10 ratios). On the contrary, except for U87 cells, AGA had no effect on the bystander effect if cell mixtures contained 50% (1/1) gemcitabine-treated cells. These results suggest that the bystander effect had a different mechanism in low versus high ratios of gemcitabine-treated cells in this experimental set-up. One likely explanation is that hENT1 could transport gemcitabine out of the treated cells making it available in the media to be picked up by untreated cells. This bystander mechanism would only be predominant at high ratios of gemcitabine-treated cells that produce a cytotoxic concentration in the cell culture medium. It is worth noting that AGA decreased the cytotoxic effect of gemcitabine in 100% treated U87 cells, and a similar reduction is also observed at the 50% cell ratio (Figure 4B).

At this point, we cannot completely discard that a cellular toxic compound triggered by gemcitabine treatment could diffuse through gap junctions and mediate the bystander effect. However, we favor the diffusion of gemcitabine and its metabolites because it resembles a well described bystander effect mechanism adopted by another nucleoside analogue, GCV [25,26]. Furthermore, two other non-nucleoside chemotherapeutic agents, cisplatin and temozolomide, do not generate a bystander effect (Figure 4C).

There are major differences between the vasculature of normal and that of malignant tissues. Blood vessels are well organized and in sufficient number to irrigate all cells in normal tissues. On the other hand, blood vessels are disorganized in tumors; they have arterio-venous shunts and incomplete vessel walls, leading to a sluggish and irregular blood flow. Cells that are away from blood vessels are not reached by effective doses of chemotherapeutic agents, a situation that cannot be mimicked in monolayer cultures [19,22]. The spheroid model used in this study is more clinically relevant in comparison to the two-dimensional culture system. This model is well established and commonly used to assess the efficacy of anticancer drugs. It displays gradients of nutrients and oxygen and cell proliferation occurs from the outer to the inner part of the spheroid [19,29,39]. As expected, cells in spheroids are more resistant to chemotherapy, and thus reflect closer the clinical situation (Figures 2 and 6) [21,35].

Hypoxia is commonly found in tumors due to the large distance that separate some neoplastic cells from the microvasculature. It is often associated with an aggressive tumor phenotype and resistance to radiation therapy and chemotherapy. Hypoxia inducible factor-1 (HIF-1) is a transcription factor expressed upon hypoxic conditions that regulates genes for their adaptation to a low oxygen environment [40]. As it has been reported in endothelial cells, HIF-1 is capable of repressing hENT1 during hypoxia [41]: thus we can presume that hENT1 is poorly expressed in tumor cells that are away from blood vessels. However, these cells could still be killed by gemcitabine if tumors display gap junctional intercellular communication. Cells close to blood vessels would pick-up gemcitabine using hENT1, and the drug-induced toxicity would diffuse to distant cells through gap junctions.

Down-regulation of connexin expression has been observed in certain tumors [42]. However, this biological phenomenon is not universal: in prostate cancer the decrease in Cx43 occurs in late stages and not in the benign stages [43]. Also, Cx26 is up-regulated in squamous cell lung carcinoma, breast cancer, and papillary and follicular thyroid cancers [44-46]. This view is supported by our recent finding that Cx43 expression is preserved in 77% of a large number of glioblastoma tumor samples, and that gap junctions are functional in primary glioblastoma cultures (unpublished data). For the other cancer types with less gap junctions, pharmacological strategies that increase connexin expression could be combined to gemcitabine treatment [47].

## Conclusion

This paper shows for the first time that the diffusion and cytotoxicity of a drug that is commonly used in cancer therapy (gemcitabine) is directly dependent on gap junction expression in tumor cells. We propose that the presence of gap junctions in tumor cells could be used to predict the responsiveness to the nucleoside analogue therapy. The results of this study may have strong implications in the clinical context of the various types of solid tumors for which gemcitabine is used alone or in combination.

## Competing interests

The authors declare that they have no competing interests.

## Authors' contributions

SC designed and carried out the experiments and wrote the initial draft of the manuscript. KG design and perform one experiment. POdeCL contributed to the experimental design and critical revision of the manuscript. MC contributed to the conception and design of the entire study and the final editing of the manuscript.

All authors read and approved the final manuscript.
